# Electronic Health Records As a Platform for Audiological Research: Data Validity, Patient Characteristics, and Hearing-Aid Use Persistence Among 731,213 U.S. Veterans

**DOI:** 10.1097/AUD.0000000000000980

**Published:** 2020-12-16

**Authors:** Gabrielle H. Saunders, Lauren K. Dillard, Oliver Zobay, John B. Cannon, Graham Naylor

**Affiliations:** 1 Manchester Centre for Audiology and Deafness, School of Health Sciences, University of Manchester, Manchester, United Kingdom; 2 VA Rehabilitation R&D, National Center for Rehabilitative Auditory Research, Portland, Oregon, USA; 3 Department of Communication Sciences and Disorders, University of Wisconsin-Madison, Madison, Wisconsin, USA; 4 Division of Clinical Neuroscience, School of Medicine, University of Nottingham, Nottingham, United Kingdom.

**Keywords:** Audiology, Comorbidity, Electronic health records, Hearing aid outcome, Hearing aid use, Hearing aids, Hearing health care, Multimorbidity, Persistence, Veterans Administration, Veterans Health Administration

## Abstract

This article presents a summary of audiological, general health, and hearing aid (HA) outcome data in a large sample of U.S. Veterans receiving HAs. The current article also provides the foundation for a series of papers that will explore relationships between a wide range of factors and HA outcomes.

**Design:**

The patient sample is all (n = 731,213) patients for whom HAs were ordered between April 2012 and October 2014 through the U.S. Veterans Health Administration Remote Order Entry System. For these patients, Veterans Affairs electronic health records (EHRs) stored in various databases provided data on demographics, received diagnostic and procedure codes (2007 to 2017), audiometry, self-reported outcomes up to 6 months postfitting, and HA battery orders (to 2017). Data cleaning and preparation was carried out and is discussed with reference to insights that provide potential value to other researchers pursuing similar studies. HA battery order data over time was used to derive a measure of long-term HA use persistence. Descriptive statistics were used to characterize the sample, comparative analyses against other data supported basic validity assessment, and bivariate analyses probed novel associations between patient characteristics and HA use persistence at 2 years postfitting.

**Results:**

Following extensive cleaning and data preparation, the data show plausible characteristics on diverse metrics and exhibit adequate validity based on comparisons with other published data. Further, rates of HA use persistence are favorable when compared against therapy persistence data for other major chronic conditions. The data also show that the presence of certain comorbid conditions (Parkinson’s disease, diabetes, arthritis, and visual impairment) are associated with significantly lower HA use persistence, as are prior inpatient admissions (especially among new HA recipients), and that increasing levels of multimorbidity, in general, are associated with decreasing HA use persistence. This is all despite the fact that deriving relevant audiological care-process variables from the available records was not straightforward, especially concerning the definition of the date of HA fitting, and the use of battery ordering data to determine long-term HA use persistence.

**Conclusions:**

We have shown that utilizing EHRs in audiology has the potential to provide novel insights into clinical practice patterns, audiologic outcomes, and relations between factors pertaining to hearing and to other health conditions in clinical populations, despite the potential pitfalls regarding the lack of control over the variables available and limitations on how the data are entered. We thus conclude that research using EHRs has the potential to be an integral supplement to population-based and epidemiologic research in the field of audiology.

## Abstract 2

Supplemental Digital Content is available in the text.

## INTRODUCTION

Electronic health records (EHRs) have been used by many medical professions to understand trends in health care provision, find connections between comorbid conditions, and determine factors influencing treatment outcomes (Casey et al. 2016). Typically, EHR-based studies leverage large sample sizes and diversity of data domains to illuminate relations and processes, which would be very costly to study through ab initio research designs. In audiology, few large-scale studies using EHRs exist. Some examples follow. Zapala et al. (2010) reviewed 1550 Medicare EHRs and determined that self-referral to audiology services (as opposed to via otology) would not pose a safety risk. Wilson & McArdle (2013) examined clinical audiometric data of almost 750,000 Veterans from the U.S. Department of Veterans Affairs (VA) audiological data repository to characterize notched audiometric configurations. Their findings presented a complex picture about the presence, depth, and symmetry of notched audiograms. Billings et al. (2018) examined data from the same source to determine the prevalence of normal hearing thresholds among 2.3 million Veterans seeking hearing health care within the VA health care system and the prevalence of abnormal clinical audiology test results in these Veterans. They determined that 10% of Veterans seeking hearing healthcare from the VA had normal pure-tone hearing thresholds and that 41% of these had some other audiological abnormality. Using data from a wider subset of the VA clinical data repository, Swan et al. (2017) described the prevalence of hearing loss and tinnitus in a cohort of 500,000 Iraq and Afghanistan Veterans with common post-deployment conditions, such as traumatic brain injury, post-traumatic stress disorder, and vertigo/dizziness. They used their findings to recommend that post-deployment conditions should be carefully considered in the planning of clinical care and beyond. Singh & Launer (2016) examined almost 61,000 patient records from a chain of private audiology clinics in the United Kingdom to determine whether hearing aid (HA) adoption among patients attending a first-time audiology visit with a significant other (SO) differed from that for patients attending the visit alone. They found greater HA adoption among those attending the visit with a SO, particularly for individuals with a mild hearing loss. They concluded that audiologists should encourage SOs to participate in the audiologic rehabilitation process. In Sweden, a unique system has been established to collect clinical and outcome data from audiology clinics and their patients across the country. This system combines features of clinical EHR and controlled outcomes research and has, for example, been able to show that self-reported outcomes are likely to vary more as a function of which clinic the patient visits than as a function of unilateral versus bilateral HA fitting (Arlinger et al. 2017).

Using clinical databases to conduct research has inherent limitations associated with the fact that the data are not collected for the purpose of research and thus are not always collected and entered in a controlled manner. In addition, verifying data validity is a major issue that demands substantial care and effort. Thus, before concluding that novel associations found in an EHR dataset reflect real effects, it is important to establish confidence in the dataset. See Dillard et al. (2020) for additional discussion of issues that can arise when using and interpreting data from EHR datasets.

This article describes the first stage of a project which exploits a large set of clinical data comprised of patients who have been fitted with HAs through the Veterans Health Administration (VHA). The data include a diverse array of diagnostic, care process, and outcome variables. Ultimately, we aim to elucidate clinically significant connections between these variables, which may have previously gone unnoticed or been inaccessible, and to verify other associations that have been indicated in smaller data samples.

We envisage that our findings can be used to provide a basis for future research on predictors of hearing care outcomes and potential ways in which hearing loss influences general health and vice versa, to develop practice recommendations with a view to development of more time and cost-effective clinical care pathways, and to illuminate potential issues and solutions specific to the exploitation of service-wide datasets in audiology.

The aims of this article are to provide a general description of the provenance of the dataset, to substantiate the overall validity and plausibility of its primary characteristics, to provide evidence that EHRs can be used for research in audiology, and to describe the platform for our future work with EHRs. The article is organized as follows. First, we describe the sources of the data, and the data cleaning and preparation processes, which led to the final dataset for analysis. Second, to provide a common background of data for subsequent papers, we describe the study sample by using summary statistics and descriptive analyses. Third, we provide some analyses examining internal and external validity and discuss the plausibility of the basic relationships found. Finally, we present some bivariate associations between hearing-related and health-related variables. These demonstrate the potential of such datasets for revealing previously unseen substantive associations. In future publications, we will report in depth on distinct relations among the data and interpret their significance for research and clinical practice.

## MATERIALS AND METHODS

This work was approved by the Institutional Review Board and the Research and Development Committee at the VA Portland Health Care System (Study #03566), as well as from Data Access Request Tracker (tracking number 2014-11-066-D-A04) and VA Patient Care Services (PCS).

### Relevant Aspects of VA Audiological Services

The service delivery context within which these VA data have arisen is necessary for understanding data structures, determining validity, and interpreting results; therefore, it is described here.

#### VA Healthcare System

The VHA is the largest integrated health care system in the United States. It provides care to over 9 million Veterans each year at over 1,200 health care facilities (https://www.va.gov/health/aboutVHA.asp). All Veterans who have served in the active military and were not dishonorably separated, as well as members of the Reserve forces or U.S. National Guard who successfully completed the period of Active-Duty to which they were called by federal order, qualify for VA health care benefits. Benefits include audiological care and HA provision. Since 1994, the VHA has used an EHR system, known as Veterans Health Information Systems and Technology Architecture, to store clinical and administrative data for patient records. The data here originate from the Veterans Health Information Systems and Technology Architecture system.

#### Audiology Services

Audiology services are provided at more than 500 VA sites of care to hundreds of thousands of patients annually (Veterans Health Administration 2018). Audiology appointments can be scheduled either following a referral from a VA medical professional or (since 2016) through self-referral. Once registered with the audiology service, individuals receive a hearing evaluation. If HAs are considered appropriate, they will be ordered, and a fitting appointment will be scheduled. Following the fitting, some clinics automatically schedule a HA follow-up visit, while other clinics require patients to initiate one if desired. This is dependent upon local policy.

If clinically indicated (VHA Directive 2008-070), HAs, related accessories, and batteries are provided to Veterans free of cost through the VA. Veterans receive a bilateral fitting unless patient preference or the hearing loss indicates otherwise. Veterans order a new supply of batteries when needed through the Denver Acquisition and Logistics Center. Batteries are shipped in quantities sufficient for 6 months of full-time HA use. This is determined according to the HA model in question and whether the Veteran has been fitted bilaterally or unilaterally.

#### Audiometry

In addition to saving audiometric data in local electronic patient records, audiologists are encouraged to enter these data using the Quality Audiology and Speech Analysis and Reporting system into a central database known as the Hearing Loss Repository.

#### Self-Reported Outcome: International Outcome Inventory for HA

VA audiologists are encouraged to administer a validated HA outcome questionnaire after each HA fitting to document the efficacy of treatment (Department of Veterans Affairs 2011). In 2011, the VA recommended use of the International Outcome Inventory for HAs (IOI-HAs; Cox et al. 2003) as the preferred HA outcome measure. The IOI-HA assesses HA outcome over the past 2 weeks on seven dimensions (use, satisfaction, benefit, residual activity limitations, residual participation restrictions, impact on others, quality of life) using a single item for each. The VA IOI-HA has an extra (8th) item for self-rated (unaided) hearing difficulty. A template for entering IOI-HA responses is available to audiologists within the Remote Order Entry System (ROES). The VA-recommended mode of administration for the IOI-HA is to mail the questionnaire to patients 30 days after their HA fitting, with a return envelope.

### Data Sources and Analysis Environment

**Fig. 1.**
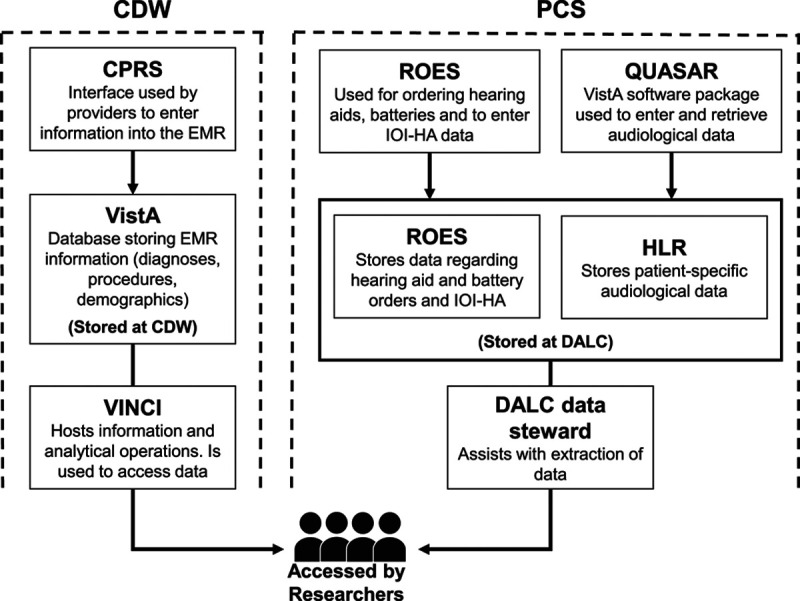
Sources of study data. CDW indicates Corporate Data Warehouse; CPRS, Computerized Patient Record System; DALC, Denver Acquisition and Logistics Center; EMR, electronic medical record; HLR, Hearing Loss Repository; IOI-HA, International Outcome Inventory for Hearing Aids; PCS, Patient Care Services; QUASAR, Quality Audiology and Speech Analysis and Reporting; ROES, Remote Order Entry System; VINCI, Veterans Affairs Informatics and Computing Infrastructure; VistA, Veterans Health Information Systems and Technology Architecture.

We obtained access to the data through two sources: (1) PCS and (2) the Corporate Data Warehouse (CDW). PCS provided access to data from ROES for information relating to device orders, battery orders and the IOI-HA, and to data from the Hearing Loss Repository relating to audiometry. The CDW provided access to demographic data and diagnostic and procedural codes. See Figure 1 for details. Every individual in the system has a unique identifier that is common across the data sources and that was used to link data extracted from each database.

All analyses were carried out within the VA Informatics and Computing Infrastructure workspace, a secure high-performance data processing platform for research. The CDW extract was provided in the form of a relational database that was accessed through Microsoft SQL Server Studio. Tables of interest were then imported into R statistical software (R Core Team 2020), where they were combined with the PCS dataset (provided as a text file) for analysis.

### Patient Sample and Data Extracts

Our aim was to extract data for all patients who had received a HA from VHA for the 31-month period from April 1, 2012, to October 31, 2014 (the “study time window”). However, because there was not a single specific Current Procedural Terminology (CPT) or Healthcare Common Procedure Coding System (HCPCS) procedural code for a HA fitting, it was necessary to extract data using a set of codes that clinical experience indicated were typically used to designate a HA fitting. Thirty-three such codes were identified (see File 1 in Supplemental Digital Content 1, http://links.lww.com/EANDH/A732 for details). Data were then extracted for any patient who had at least one of these 33 procedural codes in their outpatient CDW records within the study time window. Data from individuals in this dataset who did not have a HA order in ROES were discarded, leaving 731,213 patients who had a HA order and a procedural code indicating a HA fitting. Table 1 summarizes the variables extracted for these 731,213 individuals, including the date range, which varies depending on the type of data.

**TABLE 1.** Data tables and variables extracted from databases

Data table in dataset	Records comprised of	Source	Date range
Demographics	Patient ID, date of birth, date of death, gender	CDW	January 2007 to December 2017
Outpatient diagnoses	Patient ID, ICD code, date and time diagnostic code assigned	CDW	January 2007 to December 2017
Outpatient procedures	Patient ID, CPT/HCPCS code, date and time procedural code assigned	CDW	January 2007 to December 2017
Inpatient stays	Patient ID, admission and discharge dates, primary and secondary diagnoses (up to 25)	CDW	January 2007 to December 2017
Audiometry	Patient ID, date of examination, AC thresholds at octave and interoctave frequencies from 250 to 8000 Hz, BC thresholds at octave and interoctave frequencies from 250 to 4000 Hz	HLR	April 2012 to October 2014
Hearing aid orders	Patient ID, date of order, user type (new/experienced*); hearing aid style (BTE/ITE†/RIC); laterality (left/right/both)	ROES	April 2012 to October 2014
Battery orders	Patient ID, date of order	ROES	April 2012 to December 2017
IOI-HA	Patient ID, date of completion specified by patient, date of entry into ROES, individual item scores	ROES	April 2012 to March 2015

* Assigned by system based on the presence/absence of a prior hearing aid order in ROES.

† ITE includes all custom hearing aid styles.

AC, air conduction; BC, bone conduction; BTE, behind the ear; CDW, Corporate Data Warehouse; CPT, Current Procedural Terminology; HCPCS, Healthcare Common Procedure Coding System; HLR, Hearing Loss Repository; ICD, International Classification of Diseases; ID, identifier; IOI-HA, International Outcome Inventory for Hearing Aids; ITE, in the ear; RIC, receiver in the canal; ROES, Remote Order Entry System.

published online ahead of print December 16, 2020.

### Data Cleaning and Preparation

Since the data were extracted from clinical databases, extensive data cleaning and preparation was required before conducting any analyses. The following section describes processes undertaken to clean and prepare the data.

#### Demographic Data

In the CDW database, patients who receive care at more than one VA station have separate demographic records at each site; thus, it was necessary to identify, verify consistency, and merge these records. In total, 731,209 patients had a demographic record that included date of birth and gender, and in 99.96% of all cases, the information regarding gender and dates of birth and death was identical across records. There were 313 patients with inconsistent data for whom we used the most frequent field value across a person’s records in all analyses. Forty-two patients had a record in which the date of birth or death appeared implausible (e.g., date of death preceded the HA order). Data from these patients were excluded from analyses requiring age or survival information.

Demographic variables were used as entered in the dataset; thus, no data preparation took place.

Data from the 20.8% of patients who died between their first HA order and the end of the study period (December 31, 2017) were included or excluded in analyses depending on the time period in question. For example, to compute 2-year HA persistence rates, patients who died within 2 years after their HA fitting were excluded. In contrast, no patients were excluded for the calculation of patient demographics because all were alive at the time these data were extracted.

#### Hearing Care Processes

It was assumed that all International Classification of Diseases (ICD) and procedural codes entered into a patient’s record represented the occurrence of a valid clinical encounter; thus, no data cleaning was required here.

To reconstruct a patient’s VA-provided hearing-care history within the time frame for which data were extracted, we assembled a comprehensive list of ICD and procedural codes related to hearing care as described later and then extracted all records from the outpatient diagnosis and procedure tables that had any of these codes. All records with the same date were assumed to represent a single hearing care appointment during which multiple procedures had taken place.

Identifying all codes pertaining to hearing care (additional to the 33 deemed to indicate a HA fitting) was complex because there are over 60,000 ICD-9, ICD-10, and CPT procedural codes available in the VA system. To identify the relevant codes, we selected 100,000 random patients from our sample. For each patient, up to five dates with records of audiometry and/or HA orders in the PCS dataset were identified. For each combination of patient and date, all corresponding records of outpatient diagnoses and procedures were documented. Further codes were then added to the list following examination of the corresponding sections of the coding systems. This resulted in 158 procedural codes, 53 ICD-9 codes, and 101 ICD-10 codes, which we deemed to be “related to hearing care” (see File 2 in Supplemental Digital Content 2, http://links.lww.com/EANDH/A733). A hearing-care event was assumed to have taken place on any date on which at least one of these codes was present.

As noted earlier, there was no single procedural code for a HA fitting in the dataset. This was problematic for analyses requiring reference to the date of a HA fitting. We thus decided to designate the date of the first HA battery order as the “HA fitting date” as long it was no more than 180 days after the HA order. We consider this valid because almost all (98.6%) HA orders were followed by a battery order within 180 days and, for 62.0% of patients, the first battery order date coincided with the first hearing-care event after the HA order.

#### Audiometry

The audiometric data required extensive cleaning because there were both non-numeric values that could not be analyzed and other anomalies in the data. In File 3 (Supplemental Digital Content 3, http://links.lww.com/EANDH/A734), we describe each feature of the data that required cleaning and provide an explanation for why the anomaly arose, the solution we applied to manage the problem, and the percentage of patients with affected data. Problems encountered included the need to replace non-numeric indicators for thresholds above the upper limit of the audiometer with a value of 120 dB as recommended by Rahne et al. (2016) (23.6% of audiograms affected), and treating ambiguous non-numeric entries and values not divisible by 5 as missing (0.86% of audiograms affected). We acknowledge that both of these problems could have been managed in other ways, such as by designating a value of 5 dB greater than the frequency-specific upper limit of the audiometer, and/or by imputing missing values based on known values, and that the choice of approach will influence the outcome to some extent. However, with our large dataset and relatively few missing values, we believe the impact of our using one approach over another is minimal.

We considered an air conduction audiogram to be valid for use in analyses if, after cleaning, it had numerical threshold values (including 120 dB) at frequencies 0.5, 1.0, 2.0, and 4.0 kHz for both left and right ears. From these values, we computed left, right, and bilateral four-frequency pure-tone averages (4F-PTAs). To examine differences between left and right ears, we computed a variable to designate a clinically relevant asymmetry—defined as an absolute difference between the left and right ear 4F-PTA of ≥15 dB HL.

For simplicity in this initial article, we chose not to analyze bone conduction thresholds. Bone conduction thresholds will be addressed in future work examining associations between hearing conditions and other data.

Thirteen percent (n = 94,690) of patients had multiple valid audiograms within the 31-month study time window. These were averaged into a single 4F-PTA for analyses.

#### HA and Battery Orders

Records for HA orders and battery orders did not contain any obvious outliers or invalid entries, and missing data were not detectable; thus, these data did not require any cleaning.

Note that the ROES dataset included a variable classifying each given patient as either a new HA recipient or an experienced HA user, based on whether or not they had a prior HA order in ROES. We acknowledge that a small number of patients classified as new recipients had possibly previously used HAs that had been obtained from a source other than the VA. It was not possible to crosscheck this information using CDW data; thus, the classifications provided by the system were accepted as found. No data preparation was necessary.

#### Objective Measure of Long-Term HA Use (Persistence)

A unique feature of this study is the use of battery ordering history to provide a proxy measure of continued long-term HA use. Specifically, we considered battery orders to be analogous to prescription refills, which are often used to indirectly quantify adherence to medications. Battery order data are appropriate because 99.8% of all patients have battery order data after their first HA order.

We evaluated a variety of measures commonly used to quantify treatment adherence: (1) Compliance—which refers to the act of conforming to the recommendations made by the provider with respect to timing, dosage, and frequency of medication taking; (2) Persistence—which can be defined as the duration of time from initiation to discontinuation of therapy or the proportion of patients still continuing therapy at a given time after initiation, and (3) Medication Possession Ratio—defined as the number of doses dispensed in relation to the dispensing period, or more specifically, the ratio of the number of days for which a patient has medication on hand divided by the total number of days a patient was observed (Hess et al. 2006; Cramer et al. 2008). We chose to use persistence as our measure because it provides a straightforward description of the whole sample at a given time postfitting without being complicated by differing recommendations from individual audiologists regarding daily HA use (analogous to dose).

Details of the method used to compute persistence are described in File 4 (Supplemental Digital Content 4, http://links.lww.com/EANDH/A735). In summary, we consider “Dose” (D_dose_) to be the 6-month supply of HA batteries, the “acceptable gap” (G_acc_) in medication use (i.e., HA use) to be 12 months, and define a patient to be persistent at time T after the HA fitting if T < t_last_ + D_dose_ + G_acc_, where t_last_ is the time of the most recent battery order before T, and D_dose_ and G_acc_ are as defined earlier. HA use persistence at time T is then simply the proportion of the total patient sample who are persistent at time T.

An unavoidable limitation of using battery orders to compute persistence is that the prescribed dose period is relatively long (6 months). Thus, the minimum postfitting time T for which persistence can be <1 is 18 months. In this article, HA use persistence is always reported for T = 24 months postfitting.

#### Self-Reported Outcome: IOI-HA

The battery order-based persistence measure described earlier precludes analyses that might be informative about HA usage trends in the short-term postfitting. However, the overall dataset includes self-reported HA usage in the months following fitting.

There are no missing data or values outside of expected limits for the IOI-HA responses because the ROES system only allows entry of complete questionnaires, with entered values being constrained to integers 1 through 5.

The dataset does not explicitly link a given IOI-HA survey to a specific HA order, so we assumed that each survey reflected the outcome from the most recent preceding HA order for a given patient. The assignment of IOI-HA data to HA orders was thus unambiguous for the 99.5% of patients with a single survey (N = 157,967). There were, however, 717 patients (0.5%) with two sets of IOI-HA data, 689 of whom had two HA orders in the study time window. For the remaining 28 patients, both sets of IOI-HA data were preceded by the same HA order. For simplicity of interpretation, the IOI-HA data for these 28 patients were excluded from further analysis.

VA policy states that IOI-HAs should be returned between 14 and 180 days after a HA fitting. Only IOI-HAs returned within this time frame were used in analyses, resulting in a total of 147,285 questionnaires pertaining to 146,699 different patients.

A total IOI-HA score was computed by summing scores from items 1 to 7. Question 8 (reported unaided hearing difficulty) is analyzed separately. We acknowledge that the seven items in the IOI-HA are separate Likert scales rather than a single interval scale. However, for purposes here, we have analyzed the questionnaire as a single continuous interval scale because it facilitates comparison with results from other studies that have done likewise. Further, according to some, this approach is a pragmatic solution to a statistical controversy (see Knapp 1990 for further discussion).

#### Disease Burden

The dataset included a total of 215,342,996 outpatient ICD codes and 288,683,650 outpatient procedural codes, as well as 7,152,903 inpatient ICD codes and 1,252,359 inpatient procedural codes.

For each patient, diagnostic (ICD) and procedural (CPT/HCPCS) codes that had been assigned between January 2007 and December 2017 were extracted from the CDW database, along with time and date stamps. When deriving indices of general disease burden (see later), data concerning conditions not directly related to hearing have been taken as found, and no cleaning was conducted.

A major complication arose in the dataset because the ICD-9 code set was replaced by ICD-10 on October 1, 2015. Therefore, pre-HA order diagnoses are recorded with ICD-9 codes, while postorder diagnoses are recorded with both ICD-9 and ICD-10 codes. With the exception of codes for hearing care procedures, we only used diagnostic codes assigned before the HA order; thus, all were coded using the ICD-9 code set. For hearing care procedures, we used both ICD-9 and ICD-10 codes so we could examine hearing care both before and after the HA order.

To examine how multiple chronic conditions impact a patient’s hearing health outcomes, we created a multimorbidity index using the Chronic Condition Indicator (Healthcare Cost and Utilization Project 2016). For each ICD-9 code, the Chronic Condition Indicator indicates (1) whether the code pertains to a chronic condition or not and (2) to which of 18 body systems it belongs. The number of body systems for which a patient has at least one diagnostic code for a chronic condition is then totaled. Note that every patient in our dataset should have a code in body system 6 (“Nervous system/sense organs”) associated with their hearing loss. Therefore, in order that the multimorbidity index remains sensitive to codes for all other chronic conditions in body system 6, we computed the index following removal of hearing-loss related codes in ICD-9 code group 389.XX. The resultant multimorbidity index can range from 0 to 18. To enable a direct comparison with data from Zulman et al. (2015) (see “Disease Burden” section later), the time window used for applying diagnostic codes to the multimorbidity index was the 12 months before HA order for each individual patient, and a body system was only included in the count if the patient had at least two diagnostic codes pertaining to that system.

## RESULTS

The results presented here include descriptive analyses of the dataset, along with comparative analyses for assessment of the internal and external validity of the data, and bivariate analyses that illustrate the potential of the dataset to reveal significant and novel associations between demographic and general health variables and long-term HA use persistence.

As a brief reminder, the study sample is composed of VA patients who had a HA order between April 1, 2012, and October 31, 2014. For these patients, the following data were extracted: (1) audiometric data and HA information from April 1, 2012, to October 31, 2014; (2) battery orders from April 1, 2012, to December 31, 2017; (3) IOI-HA self-report data up to 180 days beyond each individual’s HA fitting date; and (4) demographic information, ICD, and procedural codes from January 1, 2007, to December 31, 2017.

Of the total sample of 731,213 patients with a HA order, 99.8% (n = 730,107) had one or more battery orders between April 1, 2012, and December 31, 2017, 21.7% (n = 158,684) had one or more sets of IOI-HA responses, 78.6% (n = 574,896) had air conduction audiogram data, and 18.6% (n = 136,341) had all of the above.

For a given patient, a HA order could occur at any time within the 31-month period from April 2012 to October 2014, but postorder data were extracted to December 31, 2017. Thus, the time period for which data are available after the HA order varies from patient to patient, from a minimum of 38 months (patients with a HA order at the end of October 2014) to a maximum of 69 months (patients with a HA order at the start of April 2012). In order that all patients are equitably represented in our analyses, we used a time period of 38 months post-HA order when applicable and included only those patients who survived throughout the time period in question.

### Data Descriptives

#### Patient Demographics

Of the 731,213 patients with a HA order, 53% and 47% were defined by ROES as being new HA recipients and experienced HA users, respectively. As noted earlier, an experienced user is a patient with a prior HA order in ROES.

The age range of the study sample was 20 to 90+ years. The mean age of the new recipients was 70.6 years (SD: 11.7 years) and of the experienced users was 75.7 years (SD: 11.0 years). The age distributions were not unimodal (see File 5 in Supplemental Digital Content 5, http://links.lww.com/EANDH/A736 for histograms of age at date of first HA order). Their shape reflects a combination of the onset of age-related hearing loss, variations in the number of U.S. Veterans over time, the age at which patients chose to acquire HAs, and changes in VA HA eligibility policies.

The vast majority (98.4%) of patients were male. The gender distribution was somewhat age-dependent, with 93.9% of the sample <60 years old being male, and 98.9% 60+ years old being male. A similar pattern of results is seen in the National Center for Veterans Analysis and Statistics Table 1L (https://www.va.gov/vetdata/veteran_population.asp), which documents that in 2015, 85.0% of Veterans under age 60 years were male, and 96.0% over age 60 years were male.

#### Hearing-Care Processes

A total of 9,210,309 hearing-related encounters were identified between 2007 and 2017. Overall, 53.5% were for HA-related activity only, 4.3% were for audiometry-related activity only, and 13.7% were for audiometry and HA activity combined. The remaining 28.5% of encounters were for other hearing-related issues, including tinnitus. On average, patients had 1.9 hearing-related encounters each year.

A valid audiogram was available for 570,295 patients (78.0%). To understand the timing of audiometry relative to HA orders, we examined patients with one valid audiogram and one HA order (n = 469,396). Most of these patients had a HA order immediately, or shortly after, an audiometric evaluation. Specifically, 67% of patients had a HA order on the same day as the evaluation, 14% had a HA order within 2 weeks, and 16% had a HA order between 2 weeks and 6 months after the audiometric evaluation.

#### Audiometry

**Fig. 2.**
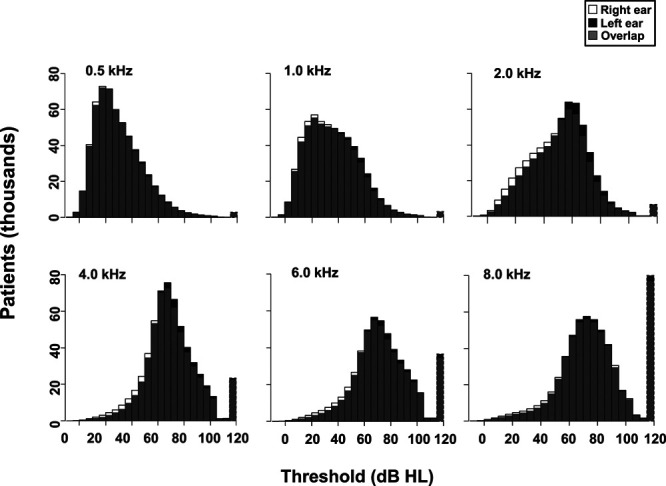
Distribution of left (black bars) and right (white bars) thresholds at 0.5 to 8.0 kHz. Gray bars indicate areas of overlap between ears. When patients had multiple audiograms, average thresholds across audiograms were used. Dashed outlines for bars at 120 dB HL denote that the value was assigned in the data cleaning/preparation process (see File 3 in Supplemental Digital Content 3, http://links.lww.com/EANDH/A734).

Among all patients with valid audiograms (n = 570,295), average 4F-PTAs were 49.9 dB (SD: 16.8 dB) and 48.7 dB (SD: 17.1 dB) for the left and right ears, respectively. A *t* test shows this to be a statistically significant difference (*t* = 67.6; *p* < 0.001). To further examine the pattern of asymmetries, we looked at the direction of asymmetry among the 14.6% of patients with a clinically relevant asymmetry. Of these, 57.7% had a greater hearing loss in the left ear (χ^2^ = 1969.7; *p* < 0.001). As seen from Figure 2, this left ear asymmetry is most evident at higher frequencies. A left ear asymmetry is not uncommon in the military (Job et al. 1998), and while a proportion of patients are likely to be left-handed, we believe the asymmetry is probably due to the impact of shooting, in which the ear closest to the barrel of the gun (left ear for right-handed shooters) tends to be worse because it is closer to the explosion, whereas the other ear is protected by the head (Yong & Wang 2015).

#### HA Orders

Table 2 shows information about the number, style, and laterality of HA orders. The vast majority of patients had one order for bilateral HAs, with almost half being for receiver in the canal devices. Within our study time window, a single HA order is to be expected, since VHA Handbook 1173.7 (Department of Veterans Affairs 2000) states that Veterans’ HAs “will be replaced when the instrument proves to be ineffective, irreparable, or the Veteran’s medical condition has changed and a different device is needed” which will rarely occur within the first 31 months (the longest time period for which we have HA order data for any patient). Additional HA orders are possible because sometimes HAs for the left and right ears are ordered separately, and because the VA permits an additional order if HAs are lost or destroyed “due to circumstances beyond the control of the Veteran,” or because the patient has severe enough hearing loss to warrant the issuance of spare HAs. This likely explains why 2% of patients had two or more HA orders. The distribution of HA styles here is comparable to that of the U.S. private sector during the same time period, where receiver in the canal, in the ears, and behind the ears represented 54%, 25%, and 21% of the market, respectively (Strom 2013).

**TABLE 2.** Characteristics of hearing aid orders in the study sample

Characteristic	No. patients	Percent (%)
No. hearing aid orders		
1	715,237	97.8
2	15,432	2.1
>2	544	0.07
Laterality of fitting		
Bilateral	680,814	91.0
Unilateral, left ear	36,602	4.9
Unilateral, right ear	30,352	4.1
Hearing aid style		
BTE	197,065	26.4
ITE*	224,081	30.0
RIC	326,622	43.7

* ITE includes all custom hearing aid styles.

BTE, behind the ear; ITE, in the ear; RIC, receiver in the canal.

#### Battery Orders

**Fig. 3.**
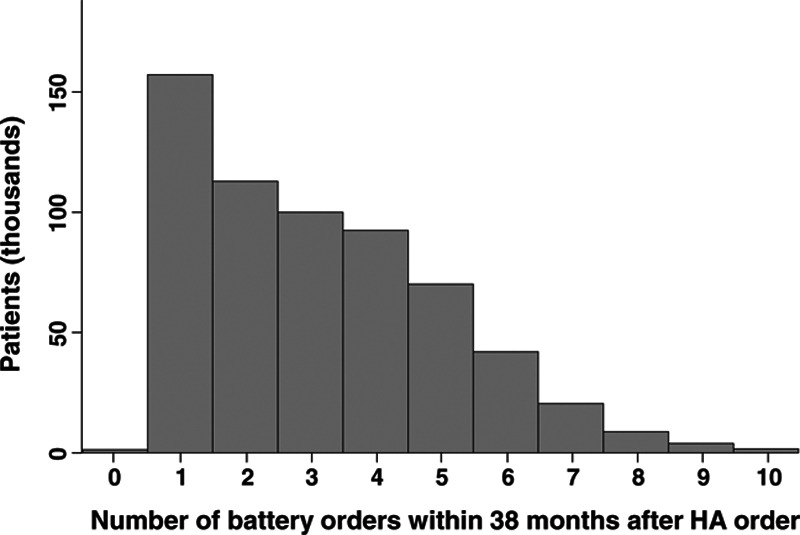
Distribution of the number of battery orders in the 38 mo following the first HA order for the 612,679 patients with a single HA order who survived at least 38 mo following the HA fitting. Data for the 0.26% of patients who had >10 HA orders are omitted from the figure. HA indicates hearing aid.

Practically all patients had at least one battery order after their first HA order; just 1785 individuals (0.24%) had none. In total, 2,806,742 battery orders were placed on or after the date of a patient’s first HA order. Figure 3 illustrates that the number of battery orders placed during the 38-month period varied widely across patients (range: 0 to 21; mean: 3.2, SD: 2.0, median: 3).

**Fig. 4.**
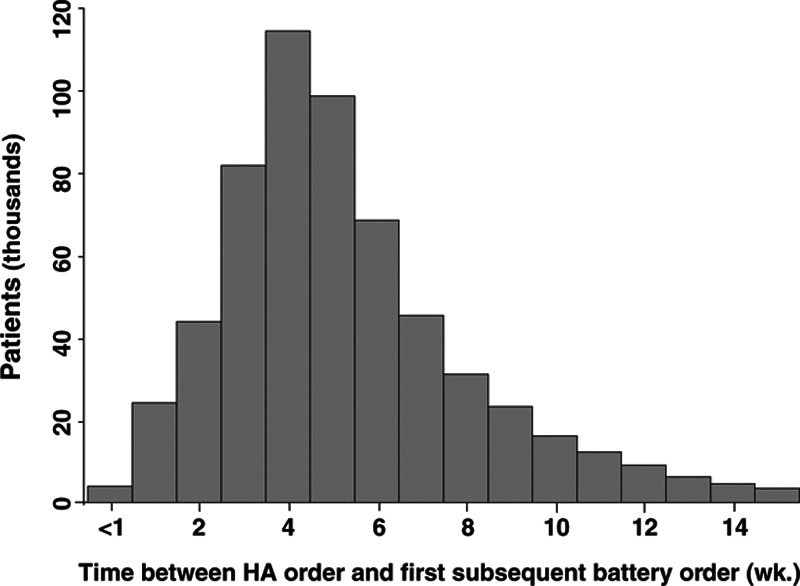
Distribution of time between the HA order and first battery order for the 612,679 patients with a single HA order who survived at least 38 mo following the HA fitting. HA indicates hearing aid.

Figure 4 shows the distribution of times between the HA order and the first battery order. The mean was 42 days (SD: 46.6 days), the median was 34 days. Based on our use of the first battery order as a proxy for the HA fitting date, we can say that on average, the HA fitting took place 42 days after the HA was ordered. The time between subsequent battery orders ranged from <1 month to 30 months (median: 7.8 months, mean: 9.5 months). This time is consistent with the 6-month period that a battery order is projected to last and perhaps indicates that patients typically use their HAs for two thirds of the time rather than fulltime.

When data are examined for individual patients, some show both long and short intervals between battery orders, perhaps suggesting variability in HA use over time.

#### HA Use Persistence

As previously described, the persistence of HA use was computed from the battery order data. The mean persistence at 24 months after the HA fitting for patients who survived for at least this time was 63.3%.

**Fig. 5.**
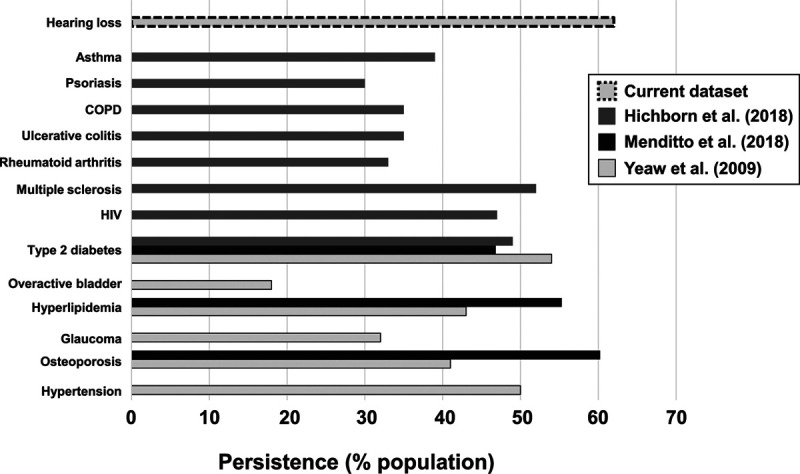
Persistence at 12 mo (%) to medications for listed conditions, along with persistence at 24 mo postfitting for hearing-aid use from this study. COPD indicates chronic obstructive pulmonary disease; HIV, human immunodeficiency virus.

Figure 5 compares this persistence value to therapy persistence for 13 other chronic conditions using data from three systematic reviews (Yeaw et al. 2009; Hichborn et al. 2018; Menditto et al. 2018). Persistence in our dataset was higher than for all other conditions, despite our value being calculated at 24 months, while the data for the other conditions is at 12 months. It is clear that relative to medications for many other chronic conditions, persistence for HA use in our sample is high.

#### Self-Reported Outcome: IOI-HA

IOI-HA data were available for 20.1% (n = 146,699) of patients. This relatively low figure is likely due to three factors, each of which plays an independent role. First, the ROES data entry system only allows for entry of complete IOI-HAs, so questionnaires returned with missing data cannot be entered. Second, administration of the IOI-HA is recommended but is not mandatory; thus, other measures, or no measures, might have been used. Third, IOI-HA data are dependent upon patients returning the questionnaire, which does not always happen. We cannot determine the extent to which each of these explanations applies.

Of the available IOI-HA surveys, 15.7% were returned within 14 to 30 days after the fitting, 54.0% were returned between 31 and 60 days, 25.9% between 61 and 120 days, and 4.5% between 121 and 180 days. Altogether, 13.8% were returned on a day for which we could also identify a HA-related appointment, suggesting that they were either completed at the appointment or returned by hand to the audiologist.

IOI-HA scores were generally high (mean: 28.8, SD: 4.1 on a scale of 7 to 35), with 64.7% of patients having a score ≥28, indicating that on average, they gave a rating of four or more on all seven questions. Only 4.5% of scores were ≤21, indicating average ratings of three or less on all items.

### Data Validity

To assess the validity of our data, we conducted analyses to examine whether expected relations between variables existed and made comparisons of the data here with other published data, as follows.

#### Representativeness of IOI-HA Data

Given that valid IOI-HA surveys were available for only 20.1% of patients, it is important to assess whether individuals with IOI-HA data differ from the rest of the study population. Welch two-sample *t* tests were used to compare age, hearing loss, and number of battery orders for patients with and without IOI-HA data, and Chi-square analysis was used to compare the proportions of new HA recipients and experienced HA users with and without IOI-HA data. The results are shown in Table 3. Relative to patients without IOI-HA data, those with IOI-HA data were on average 1.5 years older, had marginally better hearing, and ordered more HA batteries, and a higher proportion were new HA recipients. While the comparisons show statistically significant differences, the actual group mean differences are very small and likely do not demonstrate clinically meaningful differences.

**TABLE 3.** Representativeness of IOI-HA data

Characteristic	With IOI data, mean (SD)	Without IOI data, mean (SD)	Results of between-group comparisons, *t*/χ^2^ (df); *p*
Age (yr)	74.3 (10.3)	72.8 (11.9)	*t* = –48.5 (253,610); *p* < .001
4F-PTA (dB HL)	49.2 (14.8)	49.3 (15.7)	*t* = 3.32 (212,800); *p* < .001
Battery orders (over 38 mo)	3.5 (2.05)	3.1 (2.0)	*t* = –71.2 (199,560); *p* < .001
% New HA recipients	55.3	51.5	χ^2^ = 657.93 (1); *p* < .001

4F-PTA, four-frequency pure-tone average; df, degrees of freedom; HA, hearing aid; IOI, International Outcome Inventory.

The IOI-HA scores here are slightly higher than those found in other published studies for Veterans (Smith et al. 2009) and non-Veterans (Hickson et al. 2010; Arlinger et al. 2017) alike. Relative to Smith et al. (2009), the higher scores here might be associated with the improvement in technology over time. Relative to Arlinger et al. (2017) and Hickson et al. (2010), this might be because the Veterans here received HAs free of charge, while only a proportion did in the Arlinger et al. (2017) and Hickson et al. (2010) datasets. Indeed, many publications and surveys indicate that, for whatever reason, Veterans are generally more satisfied with the care they receive than are private care patients (O’Hanlon et al. 2017; Anhang Price et al. 2018).

#### Audiometric Sensitivity and Self-Reported Hearing Difficulty

**Fig. 6.**
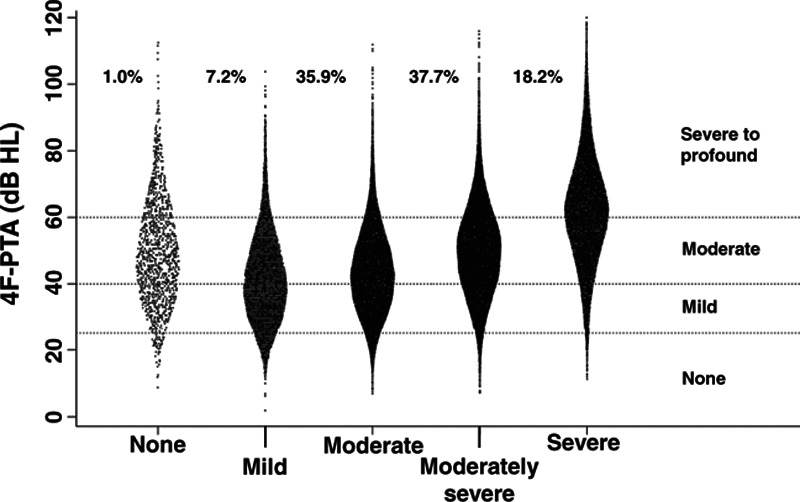
Violin plot of 4F-PTA (*y* axis) by responses to IOI-HA question 8 self-reported difficulty (*x* axis). Values indicate percentage of responses within each category. Horizontal lines indicate hearing loss severity as defined in Martin (1986). 4F-PTA indicates four-frequency pure-tone average, IOI-HA, International Outcome Inventory for Hearing Aids.

Published literature (e.g., Choi et al. 2016; Kim et al. 2017) shows a moderate association between audiometric sensitivity and self-reported hearing difficulties, we thus expect the same to hold here. To examine this, we used a violin plot to show the association between 4F-PTA and IOI-HA question 8 (Fig. 6). It illustrates that self-reported hearing loss increases as the 4F-PTA increases (Pearson *r* value: 0.41), but that there is considerable variability.

About 1% of respondents perceived no hearing difficulties despite many having moderate hearing loss or greater. Some of these individuals might have inadvertently responded to item 8 of the IOI-HA with respect to aided (rather than unaided) listening, they may have obtained HAs at the request of a family member, or they may have been confused by the IOI-HA response form. The data here do not allow us to disentangle these potential explanations.

#### Joint Distribution of Age and PTA

Table 4 compares the age versus PTA distribution in our sample with those reported by Aronoff et al. (2010) for 48,561 HA users who obtained HAs in the private sector. Regarding the overall age distribution, we see a similar proportion of individuals age <60 years in both samples, but our sample shows more young-old (60 to 69 years) and fewer old-old (80+ years). Among those <70 years old, our sample shows a much larger proportion of HA users with relatively mild losses. Apart from these differences, the age versus PTA distributions are fairly similar between the samples, reflecting age-related trends in PTA.

**TABLE 4.** Comparison of age vs. PTA distributions

Source		PTA* (dB HL)					
		<25	25–44	45–54	55–64	65–84	>84
Aronoff et al. (2010)							
% of total sample†	Age (yr)	% within age band					
12	25–59	2	30	25	19	17	7
17	60–69	1	27	26	20	20	5
28	70–79	0	21	29	27	21	3
40	80–94	0	8	24	34	30	4
Our sample							
% of total sample†	Age (yr)	% within age band					
10	25–59	14	58	17	7	4	1
33	60–69	3	44	29	15	8	1
25	70–79	1	26	31	24	16	3
31	80–94	0	9	24	32	30	5

* PTA is here calculated as mean of 0.5, 1, 2, 3, 4 kHz, consistent with Aronoff et al. (2010).

† Age bands comprising less than 1% of our sample are omitted.

PTA, pure-tone average.

The observed differences may reflect the source of payment (i.e., self-pay versus VA subsidy), as well as higher rates of HA uptake resulting from conditions associated with mild traumatic brain injury (tinnitus and central auditory processing disorders) for which HAs are being recommended in Veterans.

#### Disease Burden

**Fig. 7.**
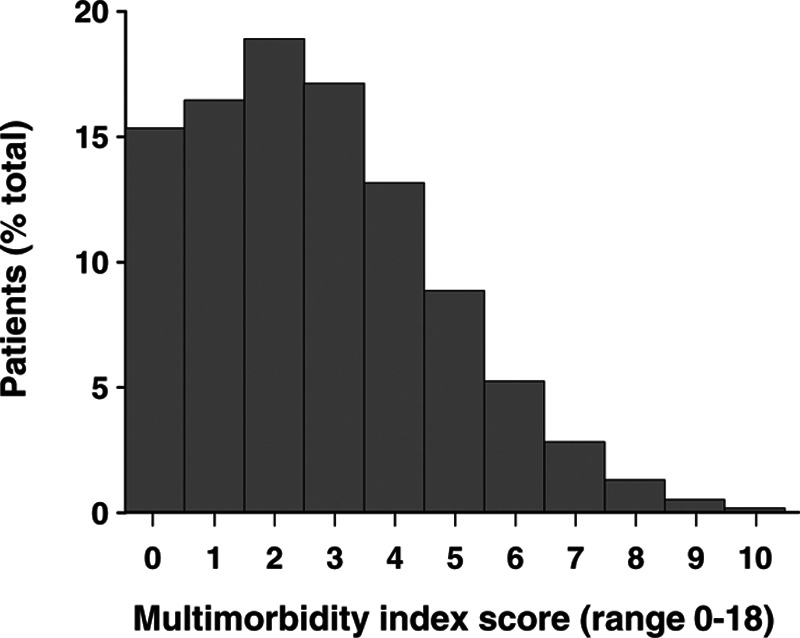
Distribution of multimorbidity index scores calculated at HA order date using a revised version of the Chronic Condition Indicator (Healthcare Cost and Utilization Project 2016) in which hearing-loss related codes in body system 6 (nervous system/sense organs) were omitted. Data for 0.06% of patients with multimorbidity scores of 11 to 13 are omitted from the figure. HA indicates hearing aid.

Figure 7 is a histogram of multimorbidity index scores at the time of the HA order. Scores ranged from 0 to 13 (mean: 2.72). Zulman et al. (2015) used the same tool to compute a multimorbidity index and reported scores for a subset of 261,699 Veterans considered “high cost” to VA, as well as for the remaining 95% of their large sample of Veterans. Among the “high cost” group by Zulman et al. (2015), 64% had ≥3 body systems affected by chronic conditions, and 18% had ≥5 body systems affected, while in the remainder of their sample, the corresponding values were 19% and 2%, respectively. In our sample, we observe 51% with ≥3 body systems affected by chronic conditions, and 20% with ≥5 body systems affected. At first glance, this appears to suggest that our sample was composed of patients with relatively high levels of multimorbidity. However, we cannot fully replicate the filtering of diagnostic counts used by Zulman et al. (2015) because of their scant reporting. As a result, the comparison with Zulman et al. (2015) neither supports nor refutes the external validity of our data. Regardless of this, we believe that the calculation of multimorbidity is valuable, both with respect to internal validity (as patients demonstrate a wide range of values) and as a robust indicator of disease burden which as seen later, provides insights into predictor variables and outcomes.

### Illustrative Associations Between Predictors and HA Outcome

The dataset allows examination of associations between predictor variables (general health, demographics, audiometry) and HA outcomes. Of the many possible such analyses, we here present those that emphasize novel associations and that focus on our measure of persistence for long-term HA use.

**Fig. 8.**
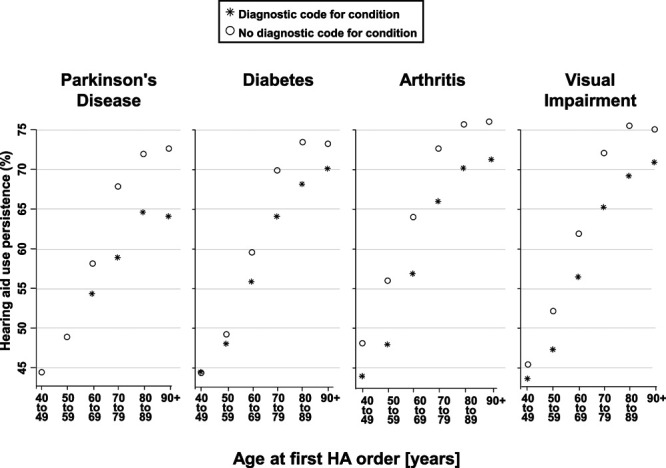
Mean HA use persistence (*y* axis) by age group (*x* axis) for individuals with (asterisk) and without (open circle) diagnostic codes assigned before the HA order indicating the presence of PD, diabetes, arthritis, and visual impairment. Data points omitted for patient groups where n < 200. HA indicates hearing aid; PD, Parkinson’s disease.

Certain chronic conditions are comorbid with hearing loss and/or auditory processing difficulties or have been suggested as conditions that may make HA use more challenging. Figure 8 shows associations between the presence of Parkinson’s disease (ICD-9-CM 332.0 and 332.1), diabetes (ICD-9-CM 250), arthritis (ICD-9-CM 360-379), and vision impairment (ICD-9-CM 710-739) at the time of the HA order and HA use persistence. Patients are separated by whether or not they had each of the four chronic conditions of interest and by age group. Figure 8 illustrates that patients with each condition had lower HA use persistence relative to those without the condition.

**Fig. 9.**
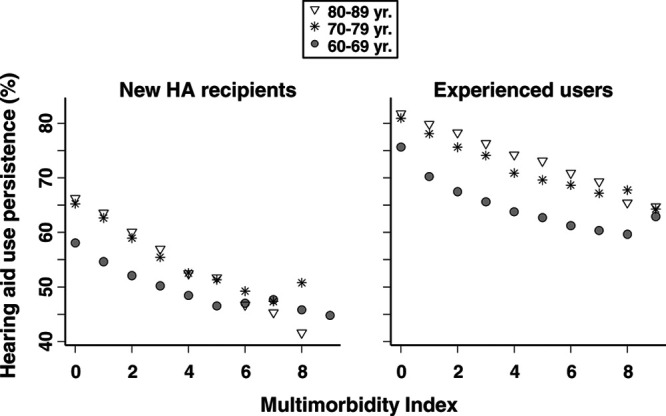
Mean HA use persistence (*y* axis) by multimorbidity index score (*x* axis) for individuals 60–69 yr (filled circle), 70–79 yr (asterisk), and 80–89 yr old (open triangle) for new HA recipients and experienced HA users separately. Data points omitted for patient groups where n < 200. HA indicates hearing aid.

**Fig. 10.**
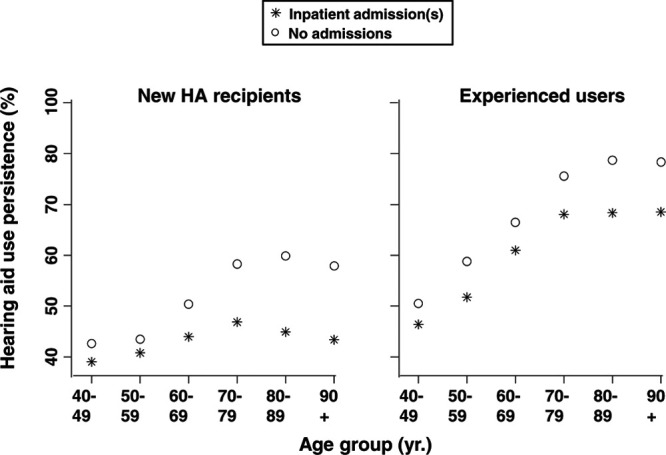
Mean HA use persistence (*y* axis) by age group (*x* axis) for individuals with (asterisk) and without (open circle) a procedural code indicating an inpatient admission before the HA order for new HA recipients and experienced HA users separately. Data points omitted for patient groups where n < 200. HA indicates hearing aid.

To examine how general disease burden impacted HA use persistence, Figure 9 shows persistence plotted against multimorbidity index score and age group, and Figure 10 shows HA use persistence for people with and without an inpatient hospitalization event before their HA order. From Figure 9, it is seen that persistence was lower for new HA recipients than experienced HA users, and lower for patients 60 to 69 years old than for older patients. Further, persistence decreased with increasing disease burden (higher multimorbidity index score), and disease burden impacted new HA recipients to a greater extent than experienced users, as illustrated by the steeper downward slopes in the former group.

Figure 10 shows similar results, with the impact of an inpatient hospitalization being greater for patients over age 70 years than for those younger than 70 years and being greater for new HA recipients than for experienced users. HA use persistence decreases slightly among the very old patients (age 90+ years). This may be explained by other variables not accounted for in these simple analyses and points to the value of multivariate approaches for in-depth understanding.

## GENERAL DISCUSSION

We have described the extraction, content, and data cleaning processes for data from a large number of patients who had received HAs from VHA within a 31-month time window. We also present findings that illustrate data validity and provide some examples of how the data can be used in audiological research. We consider that the findings can be applied to the population as a whole because while Veterans have higher prevalence of some chronic conditions relative to the general population, the behavioral and medical factors that cause these conditions are present in both, as are the mechanisms that connect conditions to hearing health and persistence of HA use.

Data cleaning and preparation involved multiple steps and processes. As further discussed in Dillard et al. (2020), these were completed to avoid biases that can arise when using EHRs for research (Verheij et al. 2018). These processes, combined with examination of internal and external validity, give us confidence in the integrity of the data. For example, we determined that

1) Application of audiological expertise (e.g., awareness that testing is conducted in 5 dB HL steps, interpreting “DNT/CNT,” (Did not test/Could not test), familiarity with the upper limit threshold of audiometers) allowed us to address anomalies associated with audiometric data.
2) Mean ages and 4F-PTAs of our sample (e.g., Aronoff et al. 2010; Abrams and Kihm 2015; Simpson et al. 2019) and mean IOI-HA scores (Smith et al. 2009; Arlinger et al. 2017) are similar to those found in other studies, as is the moderate correlation between measured and self-reported hearing loss (Choi et al. 2016; Kim et al. 2017).

We had to designate a proxy variable for the date of the HA fitting because the dataset did not include a specific CPT code for this. There were two reasonable options for a proxy HA fitting date—the date of the first battery order or the date of the first hearing care event after the HAs were ordered. We chose to use the first battery order date because 99.8% of patients had at least one valid battery order, while only 93.5% of patients had an identifiable hearing care event after the HA order. The latter is probably due to codes being assigned that were not in our hearing care code list (File 2 in Supplemental Digital Content 2, http://links.lww.com/EANDH/A733) or to instances in which the fitting was not assigned a code in the EHR. Further, 22.6% of patients had a first battery order date before the first hearing care appointment after the HA order. It seems improbable that batteries were ordered before the HA fitting, thus suggesting that the first hearing care appointment after the HA order is not always a good proxy for the HA fitting date. It is worth noting, however, that the distributions of the first HA order and the first hearing care event after the HA order are similar, they are just shifted slightly in time.

The presence of battery ordering data in our dataset provided a unique opportunity to derive a measure of ongoing HA use out to much later durations than are typically captured. Prior studies of long-term HA use are limited in population size and/or duration (e.g., Gianopoulos et al. 2002; Humes et al. 2002; Doyle et al. 2018). We used battery order information over time to compute HA use persistence at 24 months postfitting. While we acknowledge that this variable has limitations, such as the measure being coarse because battery orders are calibrated to last 6 months and the likelihood that some patients obtained HA batteries from outside of the VA, it nonetheless provides insights into long-term HA usage (up to 5 years) for a very large sample of patients, including plausible dependencies on age, user type, comorbidities, etc., as reported here.

A third variable that should be interpreted with some caution is the ROES-designated variable for HA user experience. As already noted, the ROES system labels anyone receiving their first pair of HAs from the VA as a new HA recipient. It is likely that a proportion of individuals designated as new recipients had acquired HAs in the past from a non-VA source. However, we consider the impact of this to be negligible. If anything, such mislabeling will cause outcome differences between new HA recipients and experienced HA users to be underestimated.

Missing data can cause biases that must be considered when interpreting findings. Of the 731,213 patients with a HA order, 21.4% had no audiometric data in the system, even though all must have had an audiometric examination at some point. We speculate this is either because data were not entered into the Hearing Loss Repository because this was not required before 2015 or because data were entered before our study time window. However, there is no reason to believe that such effects would result in a systematic difference between those with and without audiometric data; thus, we are not concerned about a systematic bias here. Similarly, IOI-HA data were missing for 80% of patients. As noted earlier, the reasons for this are likely to be a combination of patient response behaviors and administrative issues. Although there were statistically significant audiometric and demographic differences between individuals who had and did not have IOI-HA data, the differences are very small in magnitude and not clinically relevant, so the potential bias is unlikely to impact on the outcome of our analyses.

Not all medical history is captured in the VA EHR, as some individuals choose to seek medical care from outside of VA, some services are only provided to individuals qualifying for those services, and many Veterans have secondary health care coverage (Liu et al. 2011). However, there is no reason to expect that these differences are systematic. Further, as discussed in Dillard et al. (2020), we attempted to capture all relevant conditions by using multiple codes (as opposed to one) to classify particular diagnoses and by broadening coding categories to account for changes in coding practices over time.

Our findings of decreased HA use persistence in the presence of chronic conditions, and that the effect is greater among patients over age 70 years, and in new HA recipients, are plausible for at least two reasons. First, primary care practitioners and patients alike deprioritize hearing loss management in favor of other chronic conditions (Sidorkiewicz et al. 2019). Second, some chronic conditions may limit a patient’s physical ability to manage HAs. Specifically, vision loss leads to problems seeing small low contrast HA components, arthritis, and Parkinson’s disease impair the ability to steadily hold, insert, clean, and adjust HAs, while diabetic peripheral neuropathy and retinopathy lead to changes in sensitivity of the fingertips and to vision loss, respectively. Our findings for these specific conditions are consistent with such explanations.

The finding that these associations are stronger among older individuals could be due to the combination of disease severity and cognitive aging. Unfortunately, data regarding disease severity are unavailable from CPT codes.

There are, of course, limitations to using clinical data for research. First, clinical data are not collected or recorded in the same controlled manner as in prospective research. As a result, the data require cleaning and careful interpretation. Nonetheless, we believe that the advantages of having a vast sample size and a multitude of variables outweigh these disadvantages. The data in this particular dataset do not allow us to identify factors that led to the HA order, nor about the audiological pathways of those who did not receive HAs. In particular, we cannot determine whether some comorbid conditions prevent or impede the likelihood of a HA order in the first place. This will affect any estimation of the difficulties that the comorbid conditions present for HA usage and outcome.

In principle, the dataset does not permit firm conclusions regarding causal relationships between predictor variables and treatment outcomes. However, when consistent associations are found between diverse comorbidities and HA use persistence, and in the absence of plausible mechanisms to explain how HA use/disuse might affect these comorbid conditions, we are likely to conclude that HA use/disuse is the effect rather than the cause. Further multivariate modeling and consideration of interactions will allow deeper examination of associations between prefitting health state and postfitting outcomes and may indicate whether all associations are, in fact, the result of a common cause. In addition, where plausible hypotheses can be formulated, the quasi-longitudinal nature of the dataset will allow analyses of the impact of long-term HA use on health states postfitting.

In sum, despite these limitations, the findings have both scientific and clinical application. The availability of information about other health conditions in combination with audiological and long-term HA use data is relatively rare and allows us to consider multiple factors associated with hearing loss and HA usage. Even with these preliminary analyses, we have learned much about important associations between chronic diseases, disease burden, and HA outcome. While it has often been suggested that certain chronic diseases and disease burden will impact HA use, these findings have not been empirically illustrated before. Such findings could ultimately be applied to service planning in the same manner as did Swan et al. (2017) using VA chart records of Veterans with hearing loss and other post-deployment conditions. In addition, the use of clinical datasets such as this one facilitates the use of new methodologies in audiological research, such as machine learning and predictive modeling (Saunders et al. 2020).

Indeed, we plan to conduct further analyses of the data with a view to generating new hypotheses that could be tested prospectively using future data extracted from the same sources. This could shed light on aspects of HA outcome associated with changing hearing device technology, impacts of comorbid conditions including cognition, and changing VA practices such as telemedicine. This dataset positions us to investigate relationships of HA use, health, and additional outcome data.

## SUMMARY AND CONCLUSIONS

Research utilizing EHRs in audiology has the potential to provide novel insights into clinical practice patterns, audiologic outcomes, and relations between factors pertaining to hearing and to other health conditions in clinical populations. However, until now, large-scale data projects in the field of audiology have been rare. Here, we have described a large dataset of individuals fit with HAs in the VA system and presented findings related to internal and external data validity as well as associations between HA outcomes and other health conditions, which we believe would generalize beyond the Veteran population. The article also provides other researchers with a framework for working with EHRs and demonstrates the importance of understanding the source, integrity, and validity of the data in the EHR system. Despite the relatively uncontrolled and diverse circumstances under which the data were generated, the large size of the dataset means that the gross patterns of relations among primary variables look much as one would expect on the basis of previous literature. This suggests that the data possess acceptable validity to carry further detailed analyses. Based on this, and considering the various caveats provided, we conclude that research using EHRs has the potential to be an integral supplement to population-based and epidemiologic research in the field of audiology.

## ACKNOWLEDGMENTS

The authors thank Kevin Quitmeyer, ShienPei Silverman, Kelly Reavis, Erin Robling, and M. Patrick Feeney, for their support throughout the study.

## Supplementary Material


