# Supplementary material for: Electronic Health Records As a Platform for Audiological Research: Data Validity, Patient Characteristics, and Hearing-Aid Use Persistence Among 731,213 U.S. Veterans
Source: Ear Hear. 2020 Dec 16;42(4):927–40. doi: 10.1097/AUD.0000000000000980 (PMC8221720; doi:10.1097/AUD.0000000000000980)
Supplement: Supplementary file 1 [file aud-42-0927-s001.pdf]

CPT codes deemed to represent a HA fitting

Code	Description
97762	Checkout for orthotic/prosthetic use
98960	Education and training for patient self-management by a qualified, non-physician health care professional using a standardized curriculum, face-to-face with the patient
	Healthcare Common Procedure Coding System (HCPCS) codes
V5011	Fitting/Orientation/Checking of hearing aid
V5020	Conformity evaluation
V5050	Hearing aid, monaural, in the ear
V5060	Hearing aid, monaural, behind the ear
V5090	Dispensing fee, unspecified hearing aid
V5110	Dispensing fee, bilateral
V5130	Binaural, in the ear
V5160	Dispensing fee, binaural
V5241	Dispensing fee, monaural hearing aid, any type
V5244	Hearing aid, digitally programmable analog, monaural CIC
V5245	Hearing aid, digitally programmable analog, monaural, ITC
V5246	Hearing aid, digitally programmable analog, monaural, ITE (in the ear)
V5247	Hearing aid, digitally programmable analog, monaural, BTE
V5252	Hearing aid, digitally programmable, binaural, ITE
V5253	Hearing aid, digitally programmable, binaural, BTE
V5254	Hearing aid, digital, monaural, CIC
V5255	Hearing aid, digital, monaural, ITC
V5256	Hearing aid, digital, monaural, ITE
V5257	Hearing aid, digital, monaural, BTE
V5258	Hearing aid, digital, binaural, CIC
V5259	Hearing aid, digital, binaural, ITC
V5260	Hearing aid, digital, binaural, ITE
V5261	Hearing aid, digital, binaural, BTE
V5298	Hearing aid, not otherwise classified
V5299	Hearing service, miscellaneous
V5170	Hearing aid, CROS, in the ear
V5180	Hearing aid, CROS, behind the ear
V5200	Dispensing fee, CROS
V5210	Hearing aid, BICROS, in the ear
V5220	Hearing aid, BICROS, behind the ear
V5240	Dispensing fee, BICROS
