# Supplementary material for: Electronic Health Records As a Platform for Audiological Research: Data Validity, Patient Characteristics, and Hearing-Aid Use Persistence Among 731,213 U.S. Veterans
Source: Ear Hear. 2020 Dec 16;42(4):927–40. doi: 10.1097/AUD.0000000000000980 (PMC8221720; doi:10.1097/AUD.0000000000000980)
Supplement: Supplementary file 2 [file aud-42-0927-s002.pdf]

ICD9Code	ICD9Description
388.00	DEGENERATIVE AND VASCULAR DISORDERS, UNSPECIFIED
388.01	PRESBYACUSIS
388.02	TRANSIENT ISCHEMIC DEAFNESS
388.10	NOISE EFFECTS ON INNER EAR, UNSPECIFIED
388.11	ACOUSTIC TRAUMA (EXPLOSIVE) TO EAR
388.12	NOISE-INDUCED HEARING LOSS
388.2	SUDDEN HEARING LOSS, UNSPECIFIED
388.30	TINNITUS, UNSPECIFIED
388.31	SUBJECTIVE TINNITUS
388.32	OBJECTIVE TINNITUS
388.40	ABNORMAL AUDITORY PERCEPTION, UNSPECIFIED
388.41	DIPLACUSIS
388.42	HYPERACUSIS
388.43	IMPAIRMENT OF AUDITORY DISCRIMINATION
388.44	AUDITORY RECRUITMENT
388.45	ACQUIRED AUDITORY PROCESSING DISORDER
388.5	DISORDERS OF ACOUSTIC NERVE
388.60	OTORRHEA, UNSPECIFIED
388.61	CEREBROSPINAL FLUID OTORRHEA
388.69	OTHER OTORRHEA
388.70	OTALGIA, UNSPECIFIED
388.71	OTOGENIC PAIN
388.72	REFERRED OTOGENIC PAIN
388.8	OTHER DISORDERS OF EAR
388.9	UNSPECIFIED DISORDER OF EAR
389.00	CONDUCTIVE HEARING LOSS, UNSPECIFIED
389.01	CONDUCTIVE HEARING LOSS, EXTERNAL EAR
389.02	CONDUCTIVE HEARING LOSS, TYMPANIC MEMBRANE
389.03	CONDUCTIVE HEARING LOSS, MIDDLE EAR
389.04	CONDUCTIVE HEARING LOSS, INNER EAR
389.05	CONDUCTIVE HEARING LOSS, UNILATERAL
389.06	CONDUCTIVE HEARING LOSS, BILATERAL
389.08	CONDUCTIVE HEARING LOSS OF COMBINED TYPES
389.10	SENSORINEURAL HEARING LOSS, UNSPECIFIED
389.11	SENSORY HEARING LOSS, BILATERAL
389.12	NEURAL HEARING LOSS, BILATERAL
389.13	NEURAL HEARING LOSS, UNILATERAL
389.14	CENTRAL HEARING LOSS, BILATERAL
389.15	SENSORINEURAL HEARING LOSS, UNILATERAL
389.16	SENSORINEURAL HEARING LOSS, ASYMMETRICAL
389.17	SENSORY HEARING LOSS, UNILATERAL
389.18	SENSORINEURAL HEARING LOSS OF COMBINED TYPES, BILATERAL
389.2	MIXED CONDUCTIVE AND SENSORINEURAL HEARING LOSS

389.21	MIXED HEARING LOSS, UNILATERAL
389.22	MIXED HEARING LOSS, BILATERAL
389.8	OTHER SPECIFIED FORMS OF HEARING LOSS
389.9	UNSPECIFIED HEARING LOSS
V41.2	PROBLEMS WITH HEARING
V53.2	FITTING AND ADJUSTMENT OF HEARING AID
V72.1	EXAMINATION OF EARS AND HEARING
V72.11	ENCOUNTER FOR HEARING EXAMINATION FOLLOWING FAILED HEARING SCREENING
V72.12	ENCOUNTER FOR HEARING CONSERVATION AND TREATMENT
V72.19	OTHER EXAMINATION OF EARS AND HEARING

**ICD10Code**	**ICD10Description**
H83.3X1	Noise effects on right inner ear
H83.3X2	Noise effects on left inner ear
H83.3X3	Noise effects on inner ear, bilateral
H83.3X9	Noise effects on inner ear, unspecified ear
H90.0	Conductive hearing loss, bilateral
H90.11	Conductive hearing loss, unilateral, right ear, with unrestricted hearing on the
H90.12	Conductive hearing loss, unilateral, left ear, with unrestricted hearing on the contralateral
H90.2	Conductive hearing loss, unspecified
H90.3	Sensorineural hearing loss, bilateral
H90.41	Sensorineural hearing loss, unilateral, right ear, with unrestricted hearing on the
H90.42	Sensorineural hearing loss, unilateral, left ear, with unrestricted hearing on the
H90.5	Unspecified sensorineural hearing loss
H90.6	Mixed conductive and sensorineural hearing loss, bilateral
H90.71	Mixed conductive and sensorineural hearing loss, unilateral, right ear, with unrestricted
H90.72	Mixed conductive and sensorineural hearing loss, unilateral, left ear, with unrestricted
H90.8	Mixed conductive and sensorineural hearing loss, unspecified
H90.A11	Conductive hearing loss, unilateral, right ear with restricted hearing on the contralateral
H90.A12	Conductive hearing loss, unilateral, left ear with restricted hearing on the contralateral
H90.A21	Sensorineural hearing loss, unilateral, right ear, with restricted hearing on the contralateral
H90.A22	Sensorineural hearing loss, unilateral, left ear, with restricted hearing on the contralateral
H90.A31	Mixed conductive and sensorineural hearing loss, unilateral, right ear with restricted
H90.A32	Mixed conductive and sensorineural hearing loss, unilateral, left ear with restricted
H91.01	Ototoxic hearing loss, right ear
H91.02	Ototoxic hearing loss, left ear
H91.03	Ototoxic hearing loss, bilateral
H91.09	Ototoxic hearing loss, unspecified ear
H91.10	Presbycusis, unspecified ear
H91.11	Presbycusis, right ear
H91.12	Presbycusis, left ear
H91.13	Presbycusis, bilateral
H91.20	Sudden idiopathic hearing loss, unspecified ear

H91.21	Sudden idiopathic hearing loss, right ear
H91.22	Sudden idiopathic hearing loss, left ear
H91.23	Sudden idiopathic hearing loss, bilateral
H91.3	Deaf nonspeaking, not elsewhere classified
H91.8X1	Other specified hearing loss, right ear
H91.8X2	Other specified hearing loss, left ear
H91.8X3	Other specified hearing loss, bilateral
H91.8X9	Other specified hearing loss, unspecified ear
H91.90	Unspecified hearing loss, unspecified ear
H91.91	Unspecified hearing loss, right ear
H91.92	Unspecified hearing loss, left ear
H91.93	Unspecified hearing loss, bilateral
H93.011	Transient ischemic deafness, right ear
H93.012	Transient ischemic deafness, left ear
H93.013	Transient ischemic deafness, bilateral
H93.019	Transient ischemic deafness, unspecified ear
H93.091	Unspecified degenerative and vascular disorders of right ear
H93.092	Unspecified degenerative and vascular disorders of left ear
H93.093	Unspecified degenerative and vascular disorders of ear, bilateral
H93.099	Unspecified degenerative and vascular disorders of unspecified ear
H93.11	Tinnitus, right ear
H93.12	Tinnitus, left ear
H93.13	Tinnitus, bilateral
H93.19	Tinnitus, unspecified ear
H93.211	Auditory recruitment, right ear
H93.212	Auditory recruitment, left ear
H93.213	Auditory recruitment, bilateral
H93.219	Auditory recruitment, unspecified ear
H93.221	Diplacusis, right ear
H93.222	Diplacusis, left ear
H93.223	Diplacusis, bilateral
H93.229	Diplacusis, unspecified ear
H93.231	Hyperacusis, right ear
H93.232	Hyperacusis, left ear
H93.233	Hyperacusis, bilateral
H93.239	Hyperacusis, unspecified ear
H93.241	Temporary auditory threshold shift, right ear
H93.242	Temporary auditory threshold shift, left ear
H93.243	Temporary auditory threshold shift, bilateral
H93.249	Temporary auditory threshold shift, unspecified ear
H93.25	Central auditory processing disorder
H93.291	Other abnormal auditory perceptions, right ear
H93.292	Other abnormal auditory perceptions, left ear
H93.293	Other abnormal auditory perceptions, bilateral

H93.299	Other abnormal auditory perceptions, unspecified ear
H93.3X1	Disorders of right acoustic nerve
H93.3X2	Disorders of left acoustic nerve
H93.3X3	Disorders of bilateral acoustic nerves
H93.3X9	Disorders of unspecified acoustic nerve
H93.8X1	Other specified disorders of right ear
H93.8X2	Other specified disorders of left ear
H93.8X3	Other specified disorders of ear, bilateral
H93.8X9	Other specified disorders of ear, unspecified ear
H93.90	Unspecified disorder of ear, unspecified ear
H93.91	Unspecified disorder of right ear
H93.92	Unspecified disorder of left ear
H93.93	Unspecified disorder of ear, bilateral
H93.A1	Pulsatile tinnitus, right ear
H93.A2	Pulsatile tinnitus, left ear
H93.A3	Pulsatile tinnitus, bilateral
H93.A9	Pulsatile tinnitus, unspecified ear
Q16.9	Congenital malformation of ear causing impairment of hearing, unspecified
Z01.10	Encounter for examination of ears and hearing without abnormal findings
Z01.110	Encounter for hearing examination following failed hearing screening
Z01.118	Encounter for examination of ears and hearing with other abnormal findings
Z01.12	Encounter for hearing conservation and treatment
Z45.328	Encounter for adjustment and management of other implanted hearing device
Z46.1	Encounter for fitting and adjustment of hearing aid
Z82.2	Family history of deafness and hearing loss
Z97.4	Presence of external hearing-aid

**CPTCode**	**CPTDescription**
0208T	PURE TONE AUDIOMETRY (THRESHOLD), AUTOMATED; AIR ONLY
0209T	PURE TONE AUDIOMETRY (THRESHOLD), AUTOMATED; AIR AND BONE
0210T	SPEECH AUDIOMETRY THRESHOLD, AUTOMATED;
0211T	SPEECH AUDIOMETRY THRESHOLD, AUTOMATED; WITH SPEECH RECOGNITION
0212T	COMPREHENSIVE AUDIOMETRY THRESHOLD EVALUATION AND SPEECH RECOGNITION
69710	IMPLANTATION OR REPLACEMENT OF ELECTROMAGNETIC BONE CONDUCTION HEARING
69711	REMOVAL OR REPAIR OF ELECTROMAGNETIC BONE CONDUCTION HEARING DEVICE IN
81430	HEARING LOSS (EG, NONSYNDROMIC HEARING LOSS, USHER SYNDROME, PENDRED SYNDROME); GENOMIC SEQUENCE ANALYSIS PANEL, MUST INCLUDE SEQUENCING OF AT LEAST 60 GENES, INCLUDING CDH23, CLRN1, GJB2, GPR98, MTRNR1, MYO7A, MYO15A,
81431	HEARING LOSS (EG, NONSYNDROMIC HEARING LOSS, USHER SYNDROME, PENDRED SYNDROME); DUPLICATION/DELETION ANALYSIS PANEL, MUST INCLUDE COPY NUMBER
92502	OTOLARYNGOLOGIC EXAMINATION UNDER GENERAL ANESTHESIA
92504	BINOCULAR MICROSCOPY (SEPARATE DIAGNOSTIC PROCEDURE)
92506	EVALUATION OF SPEECH, LANGUAGE, VOICE, COMMUNICATION, AND/OR AUDITORY

92507 TREATMENT OF SPEECH, LANGUAGE, VOICE, COMMUNICATION, AND/OR AUDITORY
TREATMENT OF SPEECH, LANGUAGE, VOICE, COMMUNICATION, AND/OR AUDITORY
92508 PROCESSING DISORDER (INCLUDES AURAL REHABILITATION); GROUP, TWO OR MORE
AURAL REHABILITATION FOLLOWING COCHLEAR IMPLANT (INCLUDES EVALUATION OF
92510 AURAL REHABILITATION STATUS AND HEARING, THERAPEUTIC SERVICES) WITH OR
92550 TYMPANOMETRY AND REFLEX THRESHOLD MEASUREMENTS
92551 SCREENING TEST, PURE TONE, AIR ONLY
92552 PURE TONE AUDIOMETRY (THRESHOLD); AIR ONLY
92553 PURE TONE AUDIOMETRY (THRESHOLD); AIR AND BONE
92555 SPEECH AUDIOMETRY THRESHOLD;
92556 SPEECH AUDIOMETRY THRESHOLD; WITH SPEECH RECOGNITION
92557 COMPREHENSIVE AUDIOMETRY THRESHOLD EVALUATION AND SPEECH RECOGNITION
EVOKED OTOACOUSTIC EMISSIONS, SCREENING (QUALITATIVE MEASUREMENT OF
92558 DISTORTION PRODUCT OR TRANSIENT EVOKED OTOACOUSTIC EMISSIONS), AUTOMATED
92559 AUDIOMETRIC TESTING OF GROUPS
92560 BEKESY AUDIOMETRY; SCREENING
92561 BEKESY AUDIOMETRY; DIAGNOSTIC
92562 LOUDNESS BALANCE TEST, ALTERNATE BINAURAL OR MONAURAL
92563 TONE DECAY TEST
92564 SHORT INCREMENT SENSITIVITY INDEX (SISI)
92565 STENGER TEST, PURE TONE
92566 IMPEDANCE TESTING
92567 TYMPANOMETRY (IMPEDANCE TESTING)
92568 ACOUSTIC REFLEX TESTING, THRESHOLD
92569 ACOUSTIC REFLEX TESTING; DECAY
ACOUSTIC IMMITTANCE TESTING, INCLUDES TYMPANOMETRY (IMPEDANCE TESTING),
92570 ACOUSTIC REFLEX THRESHOLD TESTING, AND ACOUSTIC REFLEX DECAY TESTING
92571 FILTERED SPEECH TEST
92572 STAGGERED SPONDAIC WORD TEST
92573 LOMBARD TEST
92574 SWINGING STORY TEST
92575 SENSORINEURAL ACUITY LEVEL TEST
92576 SYNTHETIC SENTENCE IDENTIFICATION TEST
92577 STENGER TEST, SPEECH
92578 DELAYED AUDITORY FEEDBACK TEST
92579 VISUAL REINFORCEMENT AUDIOMETRY (VRA)
92580 ELECTRODERMAL AUDIOMETRY
92581 EVOKED RESPONSE (EEG) AUDIOMETRY
92582 CONDITIONING PLAY AUDIOMETRY
92583 SELECT PICTURE AUDIOMETRY
92584 ELECTROCOCHLEOGRAPHY
92585 AUDITORY EVOKED POTENTIALS FOR EVOKED RESPONSE AUDIOMETRY AND/OR TESTING
92586 AUDITORY EVOKED POTENTIALS FOR EVOKED RESPONSE AUDIOMETRY AND/OR TESTING

	92587	DISTORTION PRODUCT EVOKED OTOACOUSTIC EMISSIONS; LIMITED EVALUATION (TO CONFIRM THE PRESENCE OR ABSENCE OF HEARING DISORDER, 3-6 FREQUENCIES) OR
	92588	DISTORTION PRODUCT EVOKED OTOACOUSTIC EMISSIONS; COMPREHENSIVE DIAGNOSTIC EVALUATION (QUANTITATIVE ANALYSIS OF OUTER HAIR CELL FUNCTION BY COCHLEAR
	92589	CENTRAL AUDITORY FUNCTION TEST(S) (SPECIFY)
	92590	HEARING AID EXAMINATION AND SELECTION; MONAURAL
	92591	HEARING AID EXAMINATION AND SELECTION; BINAURAL
	92592	HEARING AID CHECK; MONAURAL
	92593	HEARING AID CHECK; BINAURAL
	92594	ELECTROACOUSTIC EVALUATION FOR HEARING AID; MONAURAL
	92595	ELECTROACOUSTIC EVALUATION FOR HEARING AID; BINAURAL
	92601	DIAGNOSTIC ANALYSIS OF COCHLEAR IMPLANT, PATIENT YOUNGER THAN 7 YEARS OF AGE;
	92602	DIAGNOSTIC ANALYSIS OF COCHLEAR IMPLANT, PATIENT YOUNGER THAN 7 YEARS OF AGE;
	92603	DIAGNOSTIC ANALYSIS OF COCHLEAR IMPLANT, AGE 7 YEARS OR OLDER; WITH
	92604	DIAGNOSTIC ANALYSIS OF COCHLEAR IMPLANT, AGE 7 YEARS OR OLDER; SUBSEQUENT
	92620	EVALUATION OF CENTRAL AUDITORY FUNCTION, WITH REPORT; INITIAL 60 MINUTES
	92621	EVALUATION OF CENTRAL AUDITORY FUNCTION, WITH REPORT; EACH ADDITIONAL 15
	92625	ASSESSMENT OF TINNITUS (INCLUDES PITCH, LOUDNESS MATCHING, AND MASKING)
	92626	EVALUATION OF AUDITORY REHABILITATION STATUS; FIRST HOUR
	92627	EVALUATION OF AUDITORY REHABILITATION STATUS; EACH ADDITIONAL 15 MINUTES (LIST SEPARATELY IN ADDITION TO CODE FOR PRIMARY PROCEDURE)
	92630	AUDITORY REHABILITATION; PRE-LINGUAL HEARING LOSS
	92633	AUDITORY REHABILITATION; POST-LINGUAL HEARING LOSS
	92640	DIAGNOSTIC ANALYSIS WITH PROGRAMMING OF AUDITORY BRAINSTEM IMPLANT, PER
A9040		HEARING AIDS
A9050		HEARING AID EXAM
G8565		VERIFICATION AND DOCUMENTATION OF SUDDEN OR RAPIDLY PROGRESSIVE HEARING
V5000		BASIC AUDIOLOGIC ASSESSMENT - HEARING ASSESSMENT INCLUDING THE MEASURING OF HEARING ACUITY AND TESTS RELATING TO AIR CONDUCTION, BONE CONDUCTION, RECEPTION THRESHOLD, SPEECH DISCRIMINATION, AND ACOUSTIC EMITTANCE TESTS
V5001		COMPREHENSIVE AUDIOLOGIC ASSESSMENT - HEARING ASSESSMENT INCLUDING A BASIC AUDIOLOGIC ASSESSMENT AND ASSESSMENT OF VESTIBULAR AND/OR AUDIOLOGIC
V5002		ASSESSMENT OF VESTIBULAR AND/OR AUDIOLOGIC FUNCTION BY SPECIALIZED ELECTROPHYSIOLOGIC TEST(S), EG., AUDITORY EVOKED POTENTIALS,
V5003		ASSESSMENT OF VESTIBULAR AND/OR AUDIOLOGIC FUNCTION BY SPECIALIZED
V5008		HEARING SCREENING
V5010		ASSESSMENT FOR HEARING AID
V5011		FITTING/ORIENTATION/CHECKING OF HEARING AID
V5012		COMPLETE COCHLEAR IMPLANT REHABILITATION INCLUDING ADJUSTING AND TESTING OF
V5014		REPAIR/MODIFICATION OF A HEARING AID
V5016		UNLISTED AUDIOLOGIC PROCEDURE (SPECIFY)
V5020		CONFORMITY EVALUATION
V5030		HEARING AID, MONAURAL, BODY WORN, AIR CONDUCTION
V5040		HEARING AID, MONAURAL, BODY WORN, BONE CONDUCTION

V5050	HEARING AID, MONAURAL, IN THE EAR
V5060	HEARING AID, MONAURAL, BEHIND THE EAR
V5070	GLASSES, AIR CONDUCTION
V5080	GLASSES, BONE CONDUCTION
V5090	DISPENSING FEE, UNSPECIFIED HEARING AID
V5095	SEMI-IMPLANTABLE MIDDLE EAR HEARING PROSTHESIS
V5100	HEARING AID, BILATERAL, BODY WORN
V5110	DISPENSING FEE, BILATERAL
V5120	BINAURAL, BODY
V5130	BINAURAL, IN THE EAR
V5140	BINAURAL, BEHIND THE EAR
V5150	BINAURAL, GLASSES
V5160	DISPENSING FEE, BINAURAL
V5170	HEARING AID, CROS, IN THE EAR
V5180	HEARING AID, CROS, BEHIND THE EAR
V5190	HEARING AID, CROS, GLASSES
V5200	DISPENSING FEE, CROS
V5210	HEARING AID, BICROS, IN THE EAR
V5220	HEARING AID, BICROS, BEHIND THE EAR
V5230	HEARING AID, BICROS, GLASSES
V5240	DISPENSING FEE, BICROS
V5241	DISPENSING FEE, MONAURAL HEARING AID, ANY TYPE
V5242	HEARING AID, ANALOG, MONAURAL, CIC (COMPLETELY IN THE EAR CANAL)
V5243	HEARING AID, ANALOG, MONAURAL, ITC (IN THE CANAL)
V5244	HEARING AID, DIGITALLY PROGRAMMABLE ANALOG, MONAURAL, CIC
V5245	HEARING AID, DIGITALLY PROGRAMMABLE, ANALOG, MONAURAL, ITC
V5246	HEARING AID, DIGITALLY PROGRAMMABLE ANALOG, MONAURAL, ITE (IN THE EAR)
V5247	HEARING AID, DIGITALLY PROGRAMMABLE ANALOG, MONAURAL, BTE (BEHIND THE EAR)
V5248	HEARING AID, ANALOG, BINAURAL, CIC
V5249	HEARING AID, ANALOG, BINAURAL, ITC
V5250	HEARING AID, DIGITALLY PROGRAMMABLE ANALOG, BINAURAL, CIC
V5251	HEARING AID, DIGITALLY PROGRAMMABLE ANALOG, BINAURAL, ITC
V5252	HEARING AID, DIGITALLY PROGRAMMABLE, BINAURAL, ITE
V5253	HEARING AID, DIGITALLY PROGRAMMABLE, BINAURAL, BTE
V5254	HEARING AID, DIGITAL, MONAURAL, CIC
V5255	HEARING AID, DIGITAL, MONAURAL, ITC
V5256	HEARING AID, DIGITAL, MONAURAL, ITE
V5257	HEARING AID, DIGITAL, MONAURAL, BTE
V5258	HEARING AID, DIGITAL, BINAURAL, CIC
V5259	HEARING AID, DIGITAL, BINAURAL, ITC
V5260	HEARING AID, DIGITAL, BINAURAL, ITE
V5261	HEARING AID, DIGITAL, BINAURAL, BTE
V5262	HEARING AID, DISPOSABLE, ANY TYPE, MONAURAL
V5263	HEARING AID, DISPOSABLE, ANY TYPE, BINAURAL

V5264	EAR MOLD/INSERT, NOT DISPOSABLE, ANY TYPE
V5265	EAR MOLD/INSERT, DISPOSABLE, ANY TYPE
V5266	BATTERY FOR USE IN HEARING DEVICE
V5267	HEARING AID OR ASSISTIVE LISTENING DEVICE/SUPPLIES/ACCESSORIES, NOT OTHERWISE
V5268	ASSISTIVE LISTENING DEVICE, TELEPHONE AMPLIFIER, ANY TYPE
V5269	ASSISTIVE LISTENING DEVICE, ALERTING, ANY TYPE
V5270	ASSISTIVE LISTENING DEVICE, TELEVISION AMPLIFIER, ANY TYPE
V5271	ASSISTIVE LISTENING DEVICE, TELEVISION CAPTION DECODER
V5272	ASSISTIVE LISTENING DEVICE, TDD
V5273	ASSISTIVE LISTENING DEVICE, FOR USE WITH COCHLEAR IMPLANT
V5274	ASSISTIVE LISTENING DEVICE, NOT OTHERWISE SPECIFIED
V5275	EAR IMPRESSION, EACH
V5281	ASSISTIVE LISTENING DEVICE, PERSONAL FM/DM SYSTEM, MONAURAL, (1 RECEIVER,
V5282	ASSISTIVE LISTENING DEVICE, PERSONAL FM/DM SYSTEM, BINAURAL, (2 RECEIVERS,
V5283	ASSISTIVE LISTENING DEVICE, PERSONAL FM/DM NECK, LOOP INDUCTION RECEIVER
V5284	ASSISTIVE LISTENING DEVICE, PERSONAL FM/DM, EAR LEVEL RECEIVER
V5285	ASSISTIVE LISTENING DEVICE, PERSONAL FM/DM, DIRECT AUDIO INPUT RECEIVER
V5286	ASSISTIVE LISTENING DEVICE, PERSONAL BLUE TOOTH FM/DM RECEIVER
V5287	ASSISTIVE LISTENING DEVICE, PERSONAL FM/DM RECEIVER, NOT OTHERWISE SPECIFIED
V5288	ASSISTIVE LISTENING DEVICE, PERSONAL FM/DM TRANSMITTER ASSISTIVE LISTENING
V5289	ASSISTIVE LISTENING DEVICE, PERSONAL FM/DM ADAPTER/BOOT COUPLING DEVICE FOR
V5290	ASSISTIVE LISTENING DEVICE, TRANSMITTER MICROPHONE, ANY TYPE
V5298	HEARING AID, NOT OTHERWISE CLASSIFIED
V5299	HEARING SERVICE, MISCELLANEOUS
W0125	AUDIOLOGY EXAM, VA FACILITY
W5030	AUDIOLOGY EXAM, NON-VA FACILITY
