# Supplementary material for: Electronic Health Records As a Platform for Audiological Research: Data Validity, Patient Characteristics, and Hearing-Aid Use Persistence Among 731,213 U.S. Veterans
Source: Ear Hear. 2020 Dec 16;42(4):927–40. doi: 10.1097/AUD.0000000000000980 (PMC8221720; doi:10.1097/AUD.0000000000000980)
Supplement: Supplementary file 3 [file aud-42-0927-s003.pdf]

Audiometric data cleaning (air conduction thresholds only)

Data feature	Explanation	Solution	Percentage of the 574,896 patients with AC audiograms affected
No threshold values of zero (0 dB HL) recorded in data set	Zero values presented as missing during the initial data extraction due to a coding error	Corrected by fixing the coding error and re-extracting the data	None, the problem was solved
Non-numeric values NN+, NN++ where N is a numeric value	NN+ values are typically used to indicate a situation in which the patient did not respond to the signal at the upper limit (NN) of the audiometer output at a given frequency – indicating a threshold greater than NN.	NN+ values were replaced by numeric value 120. 120 was used because it is above the upper limit of VA audiometers[1]. Its use allows us to analyse data from individuals with very severe hearing loss.	23.64%
Non-numeric values “CNT”, “DNT”	CNT or DNT is used by audiologists to mean ‘could not test’ or ‘did not test’	Values treated as missing because we cannot ascertain why the audiologist could/did not test the threshold.	0.26%
Non-numeric values NN*, +NN+, etc.	Because these values are not standard clinical abbreviations, they were assumed to be data entry errors	Values treated as missing as we cannot ascertain what they indicate	0.38%
Values not divisible by 5	Because audiometric testing is conducted in 5 dB steps, values non-divisible by 5 were assumed to be data entry errors	Values treated as missing as we cannot ascertain what they indicate	0.22%

[1] 95 dB HL for frequencies from 250 –6000Hz, 85 dB HL for 8000 Hz.
