# Supplementary material for: Electronic Health Records As a Platform for Audiological Research: Data Validity, Patient Characteristics, and Hearing-Aid Use Persistence Among 731,213 U.S. Veterans
Source: Ear Hear. 2020 Dec 16;42(4):927–40. doi: 10.1097/AUD.0000000000000980 (PMC8221720; doi:10.1097/AUD.0000000000000980)
Supplement: Supplementary file 4 [file aud-42-0927-s004.pdf]

Computation of hearing aid use persistence

In order to calculate hearing aid use persistence, it was necessary to define the period of time D_{dose} for which a ‘prescribed dose’ provides therapeutic cover. Here, this is the duration of hearing aid use which a battery order is deemed to cover. In the case of VA, a supply of batteries is calibrated to last for six months of fulltime hearing aid use, hence D_{dose} = 6 months. It is also customary to define G_{acc}, the ’acceptable gap’ in medication possession. G_{acc} is dependent on the health condition and therapy in question. We chose G_{acc} = 12 months, indicating that we considered hearing aid use to be ongoing if a new battery order takes place at most D_{dose+} $G_{acc,}$ (or 6+12=18) months after the prior battery order. While this might seem liberal, it can be considered to equate to an average of 1/3 full-time hearing aid use over the 18-month period, rather than indicating fulltime use followed by a long period of non-use.

Thus, a patient is ‘persistent’ at time T after the hearing-aid fitting if $T < t_{last} + D_{dose} + G_{acc}$ where t_{last} is the time of the most recent battery order before T, and D_{dose} and G_{acc} are as defined above.

The proportion of persistent hearing-aid users at time T after hearing-aid fitting is then the ratio of (persistent patients)/(all patients), where all patients are those for whom T is before 2017/12/31 and who have not died before T. In the main text, this proportion is termed ‘Persistence' at time T.
