# Supplementary figures and images for: Electronic Health Records As a Platform for Audiological Research: Data Validity, Patient Characteristics, and Hearing-Aid Use Persistence Among 731,213 U.S. Veterans
Source: Ear Hear. 2020 Dec 16;42(4):927–40. doi: 10.1097/AUD.0000000000000980 (PMC8221720; doi:10.1097/AUD.0000000000000980)

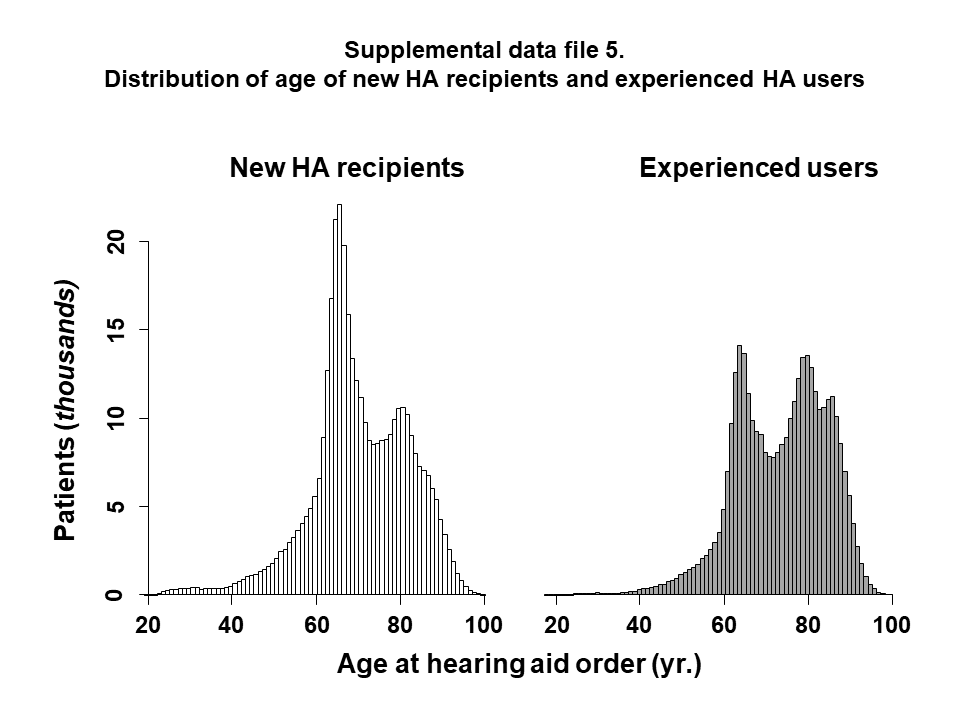

Supplement: Supplementary file 5 [file aud-42-0927-s005.tif]
